# Trends in the Breeding Population of Adélie Penguins in the Ross Sea, 1981–2012: A Coincidence of Climate and Resource Extraction Effects

**DOI:** 10.1371/journal.pone.0091188

**Published:** 2014-03-12

**Authors:** Phil O’B. Lyver, Mandy Barron, Kerry J. Barton, David G. Ainley, Annie Pollard, Shulamit Gordon, Stephen McNeill, Grant Ballard, Peter R. Wilson

**Affiliations:** 1 Landcare Research, Lincoln, New Zealand; 2 Bartonk Solutions, Nelson, New Zealand; 3 H. T. Harvey & Associates Ecological Consultants, Los Gatos, California, United States of America; 4 Antarctica New Zealand, Christchurch, New Zealand; 5 Point Blue Conservation Science, Petaluma, California, United States of America; 6 Auckland, New Zealand; Institute of Ecology, Germany

## Abstract

Measurements of the size of Adélie penguin (*Pygoscelis adeliae*) colonies of the southern Ross Sea are among the longest biologic time series in the Antarctic. We present an assessment of recent annual variation and trends in abundance and growth rates of these colonies, adding to the published record not updated for more than two decades. High angle oblique aerial photographic surveys of colonies were acquired and penguins counted for the breeding seasons 1981–2012. In the last four years the numbers of Adélie penguins in the Ross and Beaufort Island colonies (southern Ross Sea metapopulation) reached their highest levels since aerial counts began in 1981. Results indicated that 855,625 pairs of Adélie penguins established breeding territories in the western Ross Sea, with just over a quarter (28%) of those in the southern portion, constituting a semi-isolated metapopulation (three colonies on Ross Island, one on nearby Beaufort Island). The southern population had a negative per capita growth rate of −0.019 during 1981–2000, followed by a positive per capita growth rate of 0.067 for 2001–2012. Colony growth rates for this metapopulation showed striking synchrony through time, indicating that large-scale factors influenced their annual growth. In contrast to the increased colony sizes in the southern population, the patterns of change among colonies of the northern Ross Sea were difficult to characterize. Trends were similar to southern colonies until the mid-1990s, after which the signal was lost owing to significantly reduced frequency of surveys. Both climate factors and recovery of whale populations likely played roles in the trends among southern colonies until 2000, after which depletion of another trophic competitor, the Antarctic toothfish (*Dissostichus mawsoni*), may explain the sharp increasing trend evident since then.

## Introduction

The underlying factors most likely to limit the abundance of breeding seabirds in a region are prey or nesting space availability [1]. These effects are manifest in key demographic rates such as age-specific survival, dispersal and proportion breeding. This is especially so for central-place foragers like Antarctica's “true” pack-ice penguins, Emperor (*Aptenodytes forsteri*) and Adélie (*Pygoscelis adeliae*) penguins, which breed gregariously in large colonies [2]. The inability of these penguins to forage across vast distances means that they are influenced to a greater degree than volant species (e.g. albatrosses *Diomedea* spp.) by the local habitat and resources and by changes in conditions and prey stocks.

Population responses of penguins to changing ecosystems can be complex. Spatio-temporal variation in climatic variables resulting from phenomena such as long-term climate change, or shorter-term decadal atmospheric variation, i.e. factors related to the Southern Oscillation and Antarctic Oscillation (AAO, or Southern Annular Mode), are expressed through the physical environment. Examples include changes in sea-ice conditions such as concentration, extent and thickness, air temperatures, winds, sea surface temperatures (SST) and precipitation (see Ainley et al. [3] and references therein). In the case of high latitude Antarctic penguins, the issue is related to a sea-ice optimum lying between extremes that can affect them in different ways [4] (see also Jenouvrier [5] for a general application of climate optima to avian trends), as indicated empirically within the palaeoecological and ecological records of Emperor and Adélie penguin populations [6], [7], [8], [9], [10]. A switch of the AAO in the mid-1970s from variable, negative-to-positive on a decadal scale, to almost always positive thereafter brought changes in winds and sea ice [11] and in turn affected pack-ice penguin populations [3], [6], [7].

Climate change, however, by no means is the total story. Significant changes in Adélie penguin numbers could warn of changes in the abundance of their prey and/or structure and function of the marine ecosystem owing to other factors [12]. Depletion of whales and demersal fish has been associated with large-scale changes in abundance of Antarctic penguins and other diving species, i.e. seals (*Mirounga*, *Arctocephalus*) and shags (*Phalacrocorax spp*.) [13], [14], [15], [16], [17]. In the Ross Sea, the dramatic increase in Adélie penguin numbers from the mid-1970s to the early 1980s, followed by slow decline, has been correlated with the depletion by commercial whaling and then recovery of Antarctic minke whales (*Balaenoptera bonaerensis*) in International Whaling Commission Areas V and VI [18], [19]. Ross Sea penguins and minke whales feed on the same prey [20] and the penguins have since been found to begin their wintering mode in the area where most of this whaling occurred: waters north of Victoria Land [21].

Reliable repeated assessments over the long term are therefore crucial for understanding how these and other factors influence breeding population sizes and dynamics for high latitude penguins, and hence for informing management. The Adélie penguin is one of the species monitored by the Commission for the Conservation of Antarctic Marine Living Resources (CCAMLR) as part of its CCAMLR Ecosystem Monitoring Program (CEMP) to potentially detect anthropogenic effects on Antarctic marine ecosystems [22].

**Figure 1:**
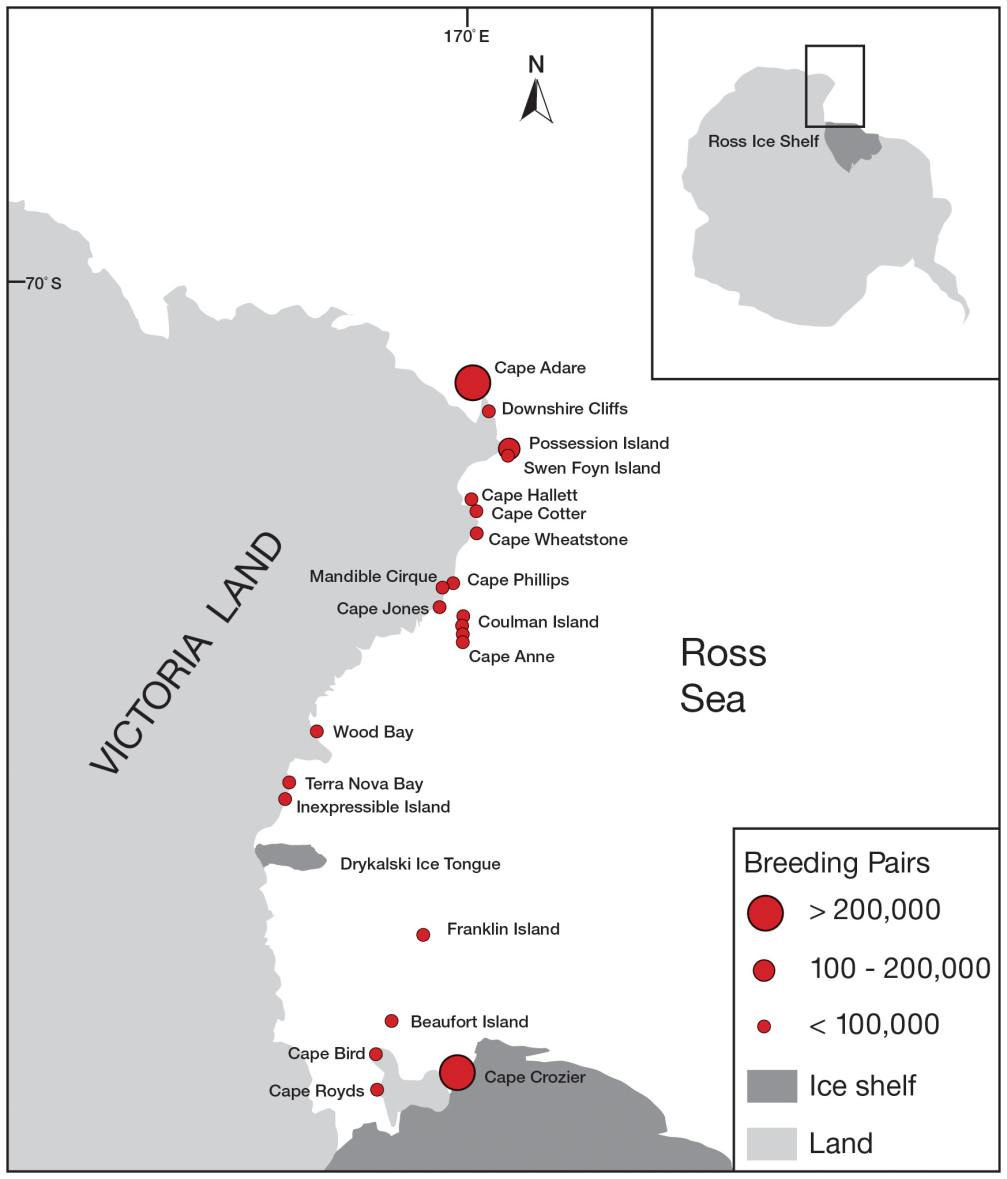
Distribution and size categories (based on 1981–2012 means) of Adélie penguin colonies from the western Ross Sea, Antarctica.

In the Ross Sea sector of the Southern Ocean, Adélie penguins breed over a latitudinal range of 1200 km, from the Balleny Islands (Buckle Island, 66° 50′ S) north of the Ross Sea to Ross Island (Cape Royds, 77° 33′ S) in the south (Fig. 1) and therefore may be exposed to a range of environmental conditions and habitats, e.g. abyssal ocean, continental slope and shelf ecosystems. The objective of this study, therefore, was to add to the long-term record and measure variation in the size of the Adélie penguin breeding populations in subregions of the western Ross Sea during the period 1981–2012. Owing to the infrequent surveys along the northern Victoria Land coast after the mid-1990s, we focused our assessment primarily on the annual or near-annual surveys of the southernmost cluster (and likely metapopulation; see [23]) of Adélie penguin colonies: three colonies on Ross Island [Cape Royds, Cape Bird (North, Middle and South), Cape Crozier (East and West)] and one on Beaufort Island, about 20 km north of Cape Bird. We examined a range of diagnostics such as means and variations in colony size, rates of change in abundance with time (annual growth rates), and evidence of trends and cycles. To conclude we offer hypotheses that might explain the trends and changes in colony sizes observed over the past three decades.

## Methods

### Census Surveys of Colonies

High angle oblique aerial photographs of Adélie penguin adults occupying nesting territories at colonies along the Victoria Land coast and offshore islands were acquired for the period 1981–2012 (see Taylor and Wilson [24](1990) and Wilson et al. [25] (2001) for discussion of trends from 1959 to 1997, based on both aerial and ground counts, as well as the discovery of 11 previously unreported breeding colonies). We define “nesting territories” as sites occupied and defended by both breeding and non-breeding adults (see below). Colonies on Ross Island (Cape Royds, Cape Bird and Cape Crozier) and Beaufort Island (Fig. 1 – herein referred to as the southern metapopulation) were photographed annually from a helicopter flying at an altitude of 2000–2500 feet (610–765 m) above ground level. Colonies along the northern Victoria Land coast (Fig. 1) were photographed only occasionally after the mid-1990s, from the open paratroop doors of a C-130 Hercules flying at a minimum altitude of 2,500 feet a.g.l. Observations from the ground showed that over-flights of helicopters and C-130 aircraft at altitudes of at least 2000 feet a.g.l. did not force birds to leave nests, though some expressed nervousness (a portion waving their flippers, P. Wilson pers. obs.; Brian Karl pers. obs.). Prior to 2006, photographs were taken with a Pentax 645 medium format black and white film-back camera. This unit was upgraded in 2006 to a Hassleblad H1D 22 MP medium-format digital camera, which was then replaced with a Canon EOS 1DS Mark III camera for the 2011/12 season.

We used photographs that were taken each year as close as possible to 1 December (range of dates: 25 November to 8 December) each season. This is a date on which the colony population, owing to its seasonal dynamics, was represented almost entirely by one member of each penguin pair incubating its eggs, and minimal numbers of non-breeders [12]. Even though our surveys were conducted within a two week window, it is possible that phenology, monitored on the ground at Ross Island colonies, still accounts for a small proportion of the inter-annual variation we observed. Census data from surveys conducted prior to 1981 were not used because they were ground-based and only for two of the three colonies – Cape Royds and Cape Bird. The population data from the years 1998–2012 are presented here for the first time. For a full description of survey methods (e.g. prioritization, location and flight approaches for colonies, and camera specifications) please refer to references [22], [24] and [25]. Operational permits were approved and issued by New Zealand's Ministry of Foreign Affairs and Trade under the Antarctic (Environmental Protection) Act 1994 and Landcare Research's Animal Ethics Committee 2005 and 2010 respectively (0509/01 and 10/09/01). Data is available upon request via: http://www.landcareresearch.co.nz/resources/data/adelie-census-data).

### Manual and Semi-automated Mapping and Counting of Colonies

Prior to 2006, film negatives were developed in a dark room, and the resulting photographs of the colony were printed and manually joined together. Once a mosaic of the colony was constructed, the appropriate photographs were enlarged and each territory occupied by a penguin counted by marking it with a dot to ensure that each was counted only once. This method of counting was slow, and it was difficult to verify counts at a later stage. However, the method had the benefit that a physical record was kept for each census.

From 2006, the colony images were captured digitally but the photographs were still processed and penguins counted manually. In 2010, semi-automatic penguin counting software was developed so that colony mapping, counts and verification of counts could occur on the computer screen [26].

### Time-Series Analysis

The Cape Bird totals are the sum of counts of 3 partitions of the colony and the Cape Crozier counts are the sum of 2 partitions of that colony, but in 3 different years counts from one of the three Cape Bird partitions were not available. To avoid biasing the total colony count towards zero these missing values for Cape Bird were interpolated by fitting a cubic smoothing spline to the colony partition time series using the “zoo” package in the statistical software “R” [27]. Variation in counts within a colony was assessed by calculating the standard deviation of the logarithmic transformation of the counts (*s*) [28].

Visual inspection of the colony time series indicated a potential change point in the trends in counts of southern colonies around the years 2000–2001. To assess differences in trends 1981–2000 vs 2001–2012, a generalized least squares model was fitted to each of the log_e_-transformed Ross Island colony counts with first-order correlation (AR(1)) in the error term to account for the inherent dependence of a count in one year on the count last year. Two models were fitted for each colony, one with a linear trend with time across all years (1981–2012), and one with different linear trends in the periods 1981–2000 and 2001–2012, in which the two periods were specified using dummy variables. The best fitting model out of the two was selected using the Akaike Information Criteria corrected for small sample sizes (AICc).

Phase plots of current colony counts against lagged counts (*t*-1 vs *t*; both log_e_-transformed) were constructed as a visual means of assessing density dependence [32]. To identify any periodicities or density dependence (Year 1 to Year 10) in the time series of colony counts, autocorrelation functions (ACF) and partial rate correlation functions (PRCF) were estimated for each of the three Ross Island colonies [29]. The significance of the correlations was tested with Bartlett's band ± 2/√*n*, where *n* is the length of time series. Spatio-temporal synchrony between annual colony growth rates (*r*_t_ = log_e_(*N*_t+1_) – log_e_(*N*_t_)) was assessed by calculating cross-correlation coefficients (*ρ*_i,j_) between all pairs of the Ross and Beaufort island colonies. The mean correlation coefficient was calculated to quantify spatial synchrony across colonies in the southern metapopulation. Because of autocorrelation in the data, the confidence intervals of the mean correlation coefficient were estimated using the bootstrap algorithm of Bjørnstad et al. [30] (1999), implemented in the “ncf” package for the statistical software “R”.

## Results

### Size and Variation in Breeding Pairs of Adélie Penguin Colonies in the Ross Sea

During our 30-year survey period 855,625 pairs of Adélie penguins on average held nesting territories just prior to hatching at colonies in the western Ross Sea (Table 1). Around a quarter (28%) of this number breed as part of the southern metapopulation with two-thirds (64%) of those birds concentrated at Cape Crozier (Table 1). The variation (or amplitude, *s*-value) in annual colony size variations across colonies in this population was similar (range = 0.11 to 0.15; Table 1). The *s*-values indicate that the average “peak to trough” ratio of counts is around one-third to one-half an order of magnitude (*s* = 0.35 is equivalent to one order of magnitude variation) indicating a lack of cycles in the data [27]. Counts of Adélie penguin breeding pairs in the northern Victoria Land population over the last 20 years have been infrequent so the variation, although also quite similar across colonies (range = 0.09 to 0.22; Table 1), needs to be interpreted with caution.

**Table 1:** Mean colony counts of nesting territories (with colony counts from Ross Island 2012 for comparison) and *s*, a measure of the amplitude of colony size variations (*n* is the total number of counts per colony), for colonies in the southern Ross Sea metapopulation and along the Victoria Land coast between 1981 and 2012.

Colony	Mean colony count (Colony counts 2012)	*s*	*N*	*n* contiguous
*Ross Island metapopulation*				
Cape Royds	2,825 (3083)	0.11	30	30
Cape Bird	43,321 (75,696)	0.13	30	30
Cape Crozier	153,632 (272,340)	0.15	28	28
Beaufort Island	39,391	0.13	17	9
*Victoria Land*				
Franklin Island West	60,540	0.11	7	2
Franklin Island East	1,110	0.12	8	2
Inexpressible Island	24,450	0.09	9	2
Terra Nova Bay	11,234	0.13	9	2
Wood Bay	1,890	0.13	8	2
Cape Anne	268	0.22	6	1
Coulman Island South	17,991	0.09	6	4
Coulman Island Middle	4,325	0.11	9	5
Coulman Island North	1,694	0.10	8	4
Cape Jones	153	0.14	6	2
Mandible Cirque	16,837	0.10	3	2
Cape Phillips	3,921	0.08	4	3
Cape Wheatstone	2,746	0.18	6	3
Cape Cotter	38,252	0.16	8	3
Cape Hallett	42,628	0.14	14	8
Foyn Island	30,494	0.12	6	1
Possession Island	111,306	0.15	3	1
Downshire Cliffs	19,617	0.12	5	2
Cape Adare	227,000	0.09	4	1

### Trends in Size of Adélie Penguin Colonies in the Ross Sea

**Figure 2:**
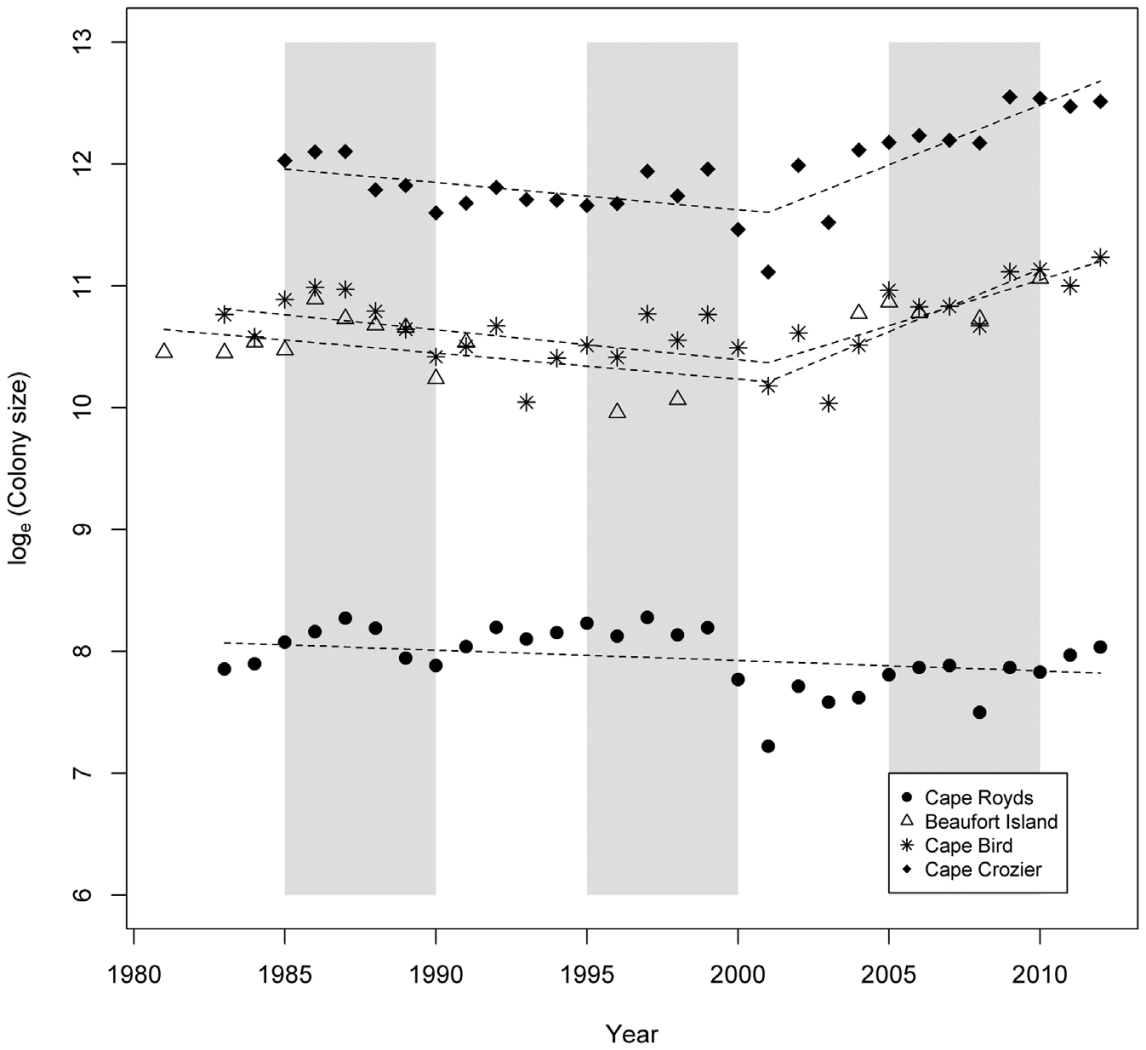
Time-series plots of the logged Adélie penguin colony counts at Cape Royds, Cape Bird, Cape Crozier and Beaufort Island, Antarctica from 1981 to 2012. The linear regression trend lines are for the period 1981–2000.

A regression model with two separate trend lines fitted the colony count data best for the Beaufort Island, Cape Bird and Cape Crozier colonies, but not for Cape Royds, where a single trend line was selected (Table 2). Our model indicated a generally decreasing trend in the number of breeding pairs at the Beaufort Island, Cape Bird and Cape Crozier colonies between 1981 and 2000, then an increasing trend from 2001 to 2012 (Table 2; Fig. 2). The small Royds colony appeared to be stable until it decreased sharply in 2000 and 2001, followed by a period of no growth or slight growth beginning in the last two years. In aggregate, this metapopulation had an average negative per capita annual growth rate of −0.019 over the 1981–2000 period followed by an average positive per capita annual growth rate of 0.067 over the 2001–2012 period, reflecting primarily the pattern in the larger colonies (not Royds). The two larger colonies had close to twice (1.8 times) the number of birds in 2012, than the 30-year average (Table 1). There was synchrony between colonies in annual changes in size through this period with a common low point in counts around Year 2001 for all colonies (Fig. 2).

**Table 2:** Summary of trend models for Cape Royds, Cape Bird and Cape Crozier (Ross Island) and Beaufort Island colony counts (log_e_-transformed) with predictor units in years from 1981 to 2012.

Colony	Best model (1 or 2 linear trends)	Trend 1981–2000 slope estimate (std error estimate)	Trend 2001–2012 slope estimate (std error estimate)
Cape Royds	1	−0.0085 (0.00797)	
Cape Bird	2	−**0.0245 (0.00862)**	**0.0755 (0.01523)**
Cape Crozier	2	−**0.0223 (0.00784)**	**0.0981 (0.01213)**
Beaufort Island	2	−0.0214 (0.01357)	**0.1028 (0.03318)**

Values in bold text indicate statistical significance of the estimated slope parameter (r = per capita annual growth rates) at *P*<0.05.

**Figure 3:**
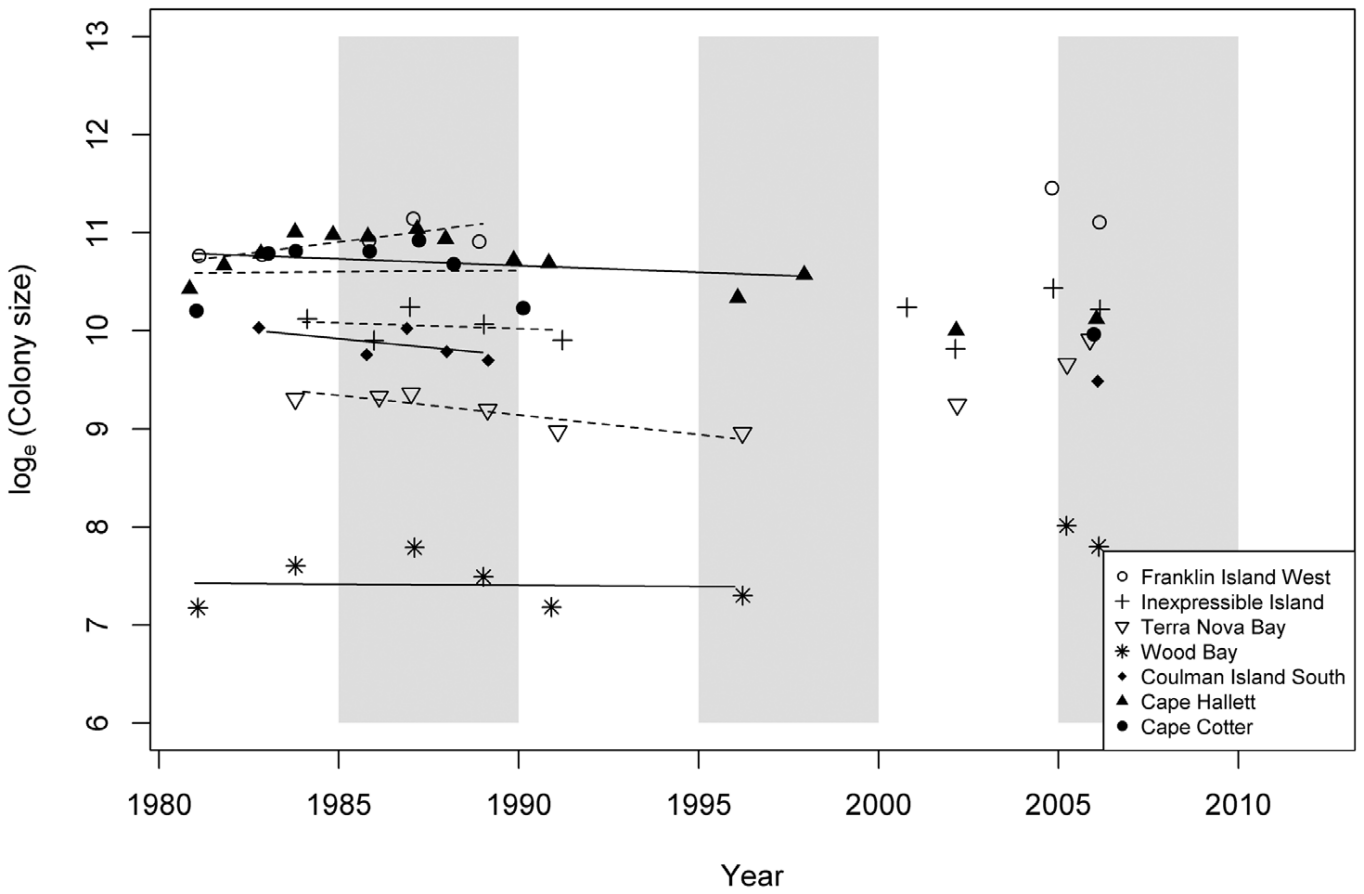
Time series plots of the logged Adélie penguin breeding pair counts from colonies that were surveyed more than five times between the years 1981 and 2012 along the Victoria Land coast, Antarctica. The linear regression trend lines are for the period 1981–2000 (in accord with Fig. 2), after which counts became too infrequent to fit a regression.

Changes in abundance of Adélie penguins at seven colonies of northern Victoria Land for which we had data from six or more surveys over the 30 years were variable. There appeared to be initial stability or perhaps decrease through 1991, but thereafter, with sparse counts, no clear trend was evident (Table 3; Fig. 3).

**Table 3:** Summary of linear trend models for northern Victoria Land colony counts (log_e_-transformed) for the years 1981–2000.

Colony	Trend/slope estimate (SE est.)	*p*-value
**Franklin Island West**	**0.0461 (0.01009)**	**0.020**
Inexpressible Island	−0.0113 (0.02224)	0.647
**Terra Nova Bay**	−**0.0400 (0.00897)**	**0.011**
Wood Bay	−0.0023 (0.02478)	0.932
Coulman Island South	−0.0361 (0.02113)	0.186
Cape Hallett	−0.0137 (0.01851)	0.478
Cape Cotter	0.0028 (0.04761)	0.955

Values in bold text indicate statistical significance of the estimated slope parameter (r = per capita annual growth rates) at *P*<0.05.

### Colony Size Regulation

**Figure 4:**
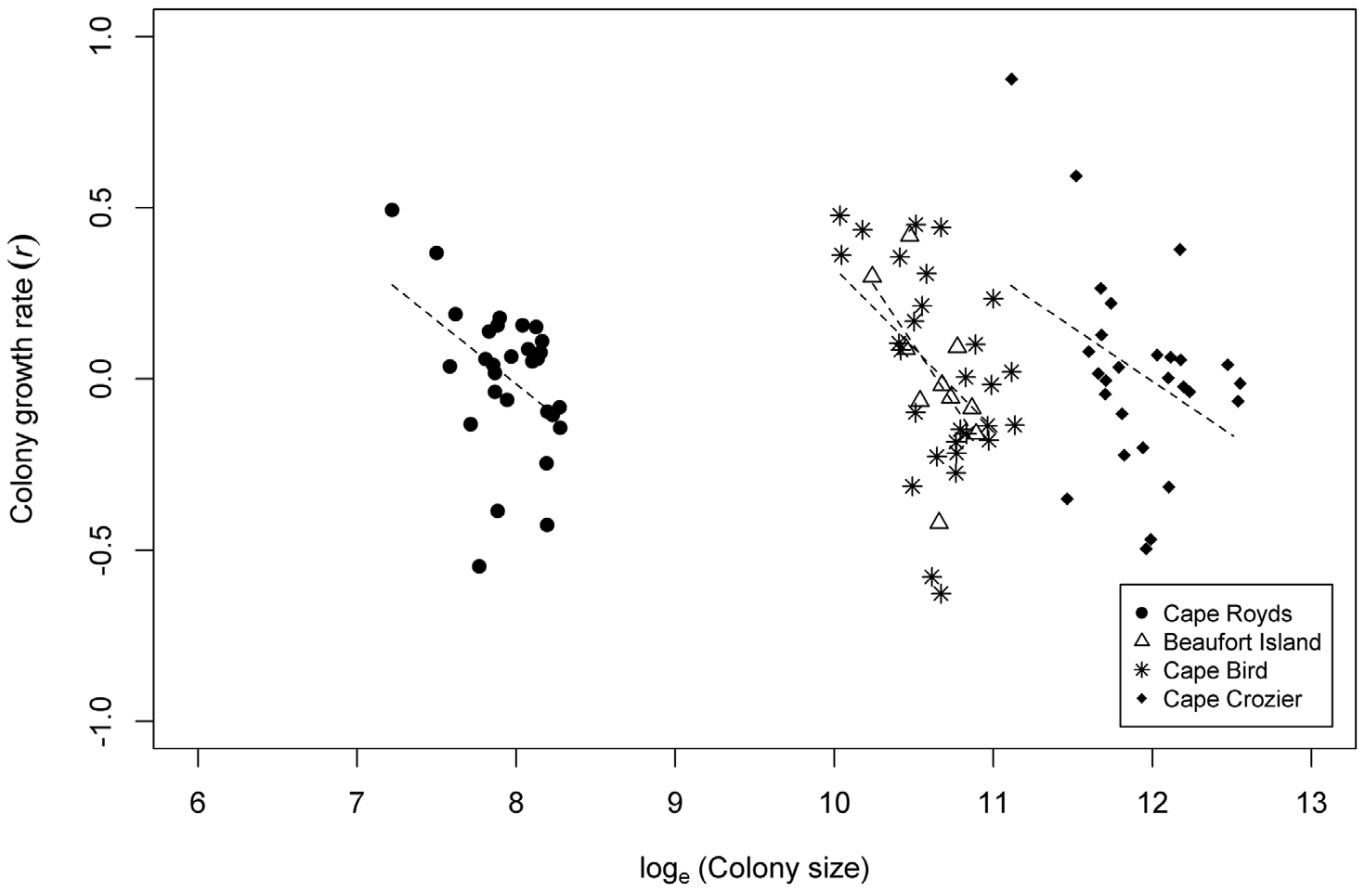
Annual colony growth rate against colony size at Cape Royds, Cape Bird, Cape Crozier and Beaufort Island, Antarctica between 1981 and 2012. A fitted linear trend is indicated by the dashed lines (fitted using ordinary least squares regression).

The auto-correlation functions (ACF) for log_e_-transformed colony sizes showed no evidence of cycles in the time series, with significant lags occurring only at 1 year or both years 1 and 2, though the length of the time series is relatively short for such detection. The slow decay of the ACF to negative values at high lags is indicative of a trend in the data (as discussed in the previous section). The partial rate correlation functions (PRCF) for the rates of change versus log_e_-transformed colony size all showed a significant negative correlation at lag 1 indicative of direct density dependence in the number of pairs returning to the colony each year. This indicates that high colony counts in one year tend to result in lower growth rates to the following year, and vice versa: low colony counts in one year tend to result in higher growth rates to the following year (Fig. 4). There were also significant negative correlations between rates of change and colony size 8 years previously for Cape Royds and Cape Crozier. Phase plots for Cape Royds and Cape Crozier showed relatively small back-and-forth fluctuations about the mean colony size throughout the 1980s and 1990s, indicative of direct density dependence, followed by larger variation in colony sizes from the Year 2000 on, indicating looser regulation. The phase plot for Cape Bird showed consistent clockwise orbits, indicative of ongoing delayed density-dependent feedback on colony size.

### Synchrony in Annual Colony Growth Rates

**Figure 5:**
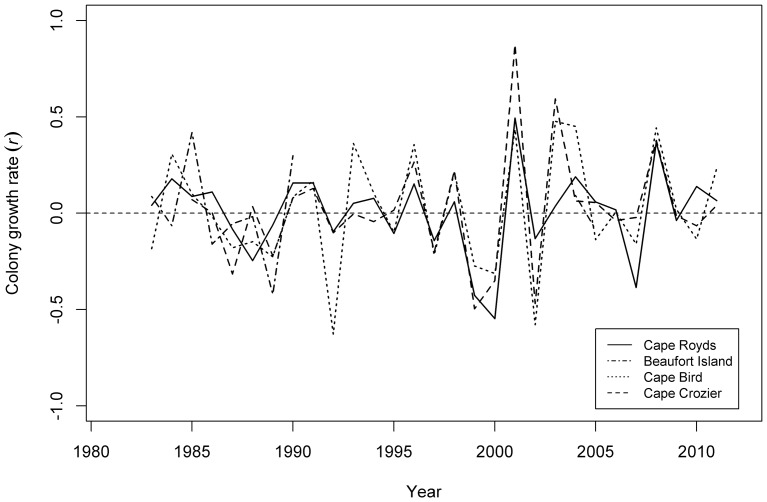
Annual growth rates for Adélie penguin colonies at Cape Royds, Cape Bird, Cape Crozier and Beaufort Island, Antarctica from 1981 to 2012.

Annual colony growth rates (*r*) for the southern population showed a striking level of synchrony through time (Fig. 5). This was confirmed by a high mean (cross-) correlation for the Ross and Beaufort island colonies of 0.59, with a 95% (bootstrapped) confidence interval of 0.35–0.77. The lowest levels of synchrony were between Beaufort Island and the other colonies (*R* = 0.35–0.59). Synchrony between the other three colonies (Cape Royds, Cape Bird and Cape Crozier) was higher, with correlation coefficients of 0.68–0.77.

## Discussion

### Status and Trends in the Ross Sea Adélie Penguin Population

The aerial survey data reported here provide the most recent counts of Adélie penguin breeding pairs in the Ross Sea, extending in time by 2.5 decades one of the longest monitored Antarctic penguin populations. In the last four years, the numbers of Adélie penguins, at least in the southern Ross Sea, have grown to their highest levels since aerial counts began (1981), as well as since all counts began in 1959 (cf. [23]). Even so, our mean count of 855,625 breeding pairs for the Ross Sea was still lower than the mean count of 938,877 breeding pairs reported by R.H. Taylor during the 1980s (pers. comm. in Woehler [31] 1993). This difference can be potentially explained by the infrequent surveys of Victoria Land colonies, where in the past approximately 75% of the Ross Sea population have bred, over the last 25 years when numbers were potentially substantially lower than in the 1980s. We do not report numbers from the four Victoria Land colonies west of Cape Adare (Fig. 1) (no more than ∼3,000 pairs [12], Lyver unpubl. data).

The most current assemblage of counts indicates about 38% of the circum-Antarctic Adélie penguin population breeds in the Ross Sea [12]. Coupled with observed increases in the Ross Sea over the last decade, expanding distributions in east Antarctica [32], and decreases in colonies in the northern Antarctic Peninsula region but increases in the south [16], this percentage may still be approximately correct. A large concentration (historically ∼47% of the Ross Sea population) in the extreme northern Victoria Land focuses around four colonies: Possession Island, Foyn Island, Downshire Cliffs, and Cape Adare (Fig. 1). We have limited information about recent trends in these colonies, although some of them might be space-limited. It is likely that these large concentrations occur where they do because of nesting habitat availability, proximity to persistent polynyas where open water facilitates access to prey [12], and proximity to the highly productive Ross Sea Slope Front [33].

The presence of unoccupied sub-colony mounds with ample supplies of pebbles for nest-building indicates that the colonies on Ross Island are not space-limited. However, recent recession of ice fields on Beaufort Island have resulted in more nesting habitat, meaning that recruits within this metapopulation no longer have to emigrate to the Ross Island colonies to find nesting space [23]. Therefore, only recently has Beaufort been able to grow. We suggest that the numbers breeding in the southern colonies are now more likely to be limited by food accessibility, mediated in part by environmental stochasticity (especially fast ice cover) in the case of Cape Royds and perhaps Cape Bird [34] and by inter- and intra-specific competition at Cape Crozier and Beaufort Island [20], [23], [35].

### Drivers of Adélie Penguin Abundance in the Ross Sea

The similar low level of amplitude associated with colony size variation across the southern metapopulation indicates that colonies could be responding in concert to a single or multiple common drivers (except for Royds during and after the mega-iceberg B-15 era; see below). This is supported by the high level of synchrony and correlation in annual growth (Fig. 5). The relationship between annual colony growth rates and size indicates that a form of density dependence could be influencing colonies, supported by the fact that initially recent growth at Beaufort was due to infilling of the colony followed by expansion into new breeding habitat [33]. Since currently there is ample space for additional growth at all these colonies (see [23]), competition for food or food availability is the most likely variable affecting birds' body condition and subsequent survival or decision whether to return to breed in the following year.

### Effects of Physical Changes in the Penguins' Environment

Among the possible common drivers is sea-ice variation, with the mega-icebergs showing this quite well at the local, mesoscale. The depression in abundance of Adélie penguins in the southern Ross Sea population in 2001 was a common low point coinciding with the arrival of the giant icebergs B-15A and C-16 in January 2001, and which remained in place until winter 2005. These icebergs, as well as B-15B, resulted in a one-season reduction in primary production in the Ross Sea polynya, 2000/01 [36], [37], as well as an alteration of surface circulation [38]. The calving of an even larger iceberg in 2002/03, C-19, also led to a one-season decrease in production (owing to more sea ice, and less ocean exposed to sunlight; [39], C. Smith, unpubl. data). While grounded, B-15A and C-16 prevented the wind from blowing sea ice from the southwestern Ross Sea northward, except in 2003 when winds were particularly strong. Lots of sea ice decreased access to the ocean and food [4], [9].

Many Adélie penguin adults failed or abandoned breeding attempts early in the breeding season in the initial year of the B-15A and C-16 iceberg groundings (2001/02). In the case of Cape Royds, conditions were especially daunting with 70 km of fast ice remaining in place in McMurdo Sound for most of the period 2001–2005; besides nest desertions, many adults eventually emigrated to Cape Bird [40]. Thus, recovery of the Cape Royds colony has been slow, showing positive signs only in the last couple of years. In contrast, the colonies at Beaufort, Bird and Crozier, which were less affected by the increased sea ice, except in the initial iceberg year, subsequently grew throughout the remaining mega-iceberg era up to the present.

The effects of ice bergs and the altered sea-ice regime were not confined just to the southern Adélie penguins. B-15A and C-16 presence resulted in an initial decrease in Beaufort Island and Cape Crozier emperor penguins, followed by slow recoveries still ongoing [41], [42] (Ballard et al. unpubl. data). The extensive fast ice that remained in McMurdo Sound 2001–2005 resulted in a depression in Weddell seal (*Leptonychotes weddelli*) numbers, but which recovered entirely once the icebergs departed and annual fast ice returned [43].

Sea-ice cover at the larger scale can also affect Adélie penguins in a number of ways. A previous study analysing trends up to 1998 demonstrated a correlation between declines in the southern colonies 4–5 years after anomalously extensive sea ice in the Ross Sea sector of the Southern Ocean [25]. Those authors hypothesized that juvenile Adélie penguin survivorship decreased in years when extensive sea ice carried the penguins well beyond the productive feeding grounds that lie south of the Southern Boundary of the Antarctic Circumpolar Current (SBACC). If the winter-sea-ice extent in the Ross Sea sector regularly extended beyond the SBACC then natural growth rates of Adélie penguin colonies could be affected, given that sea-ice extent has been growing in the Ross Sea sector, including more years of ice extending north of the SBACC [44]). The sea-ice season and large-scale extent in the Ross Sea region has increased by 3 months and thousands of square kilometres in the past 30 years, with most of the increase in season occurring in the western Ross Sea slope area, i.e. what is known as the Ross Passage Polynya (cf. [44], [45]). This trend, owing to increasing winds associated with the AAO switch, is projected to continue over the next few decades [9], [11], [44]. At the same time, the latent-heat, wind-driven polynyas of the Ross Sea have become more persistent, i.e. their sea-ice season is decreasing [46], which facilitates penguin foraging during the breeding season at least to a point, after which further polynya growth is neutral to penguin well-being.

### Effects of Biological Changes in the Penguins' Environment

The more favorable polynya behaviour also likely played a complex role in trends through the 1990s [3]. By the 1990s, polynya prevalence had reached a point at which further increase would not affect the penguins, and the slow population decrease in the 1990s could be related to recovery of the minke whale population from whaling in the 1970 and early 1980s (see Introduction: reason for the 1970s–80s increase in penguins). The fact that the penguin decrease did not reach the low levels exhibited before the whaling we ascribe to climate effects and, namely, increasing persistence of coastal polynyas that favoured colony growth.

If the earlier study by Wilson et al. [25] (2001) is correct–decreases in colonies happen following years of extensive sea ice in the Ross Sector–then Adélie penguin numbers in the Ross Sea should be decreasing (cf. in accord with increasing ice extent and season [44]). The obvious question is why is this not happening? We propose that the large increases observed in the southern population over the last decade, as with the whales earlier, might be the result of competitive release. Owing to the recent disappearance of another trophic competitor in the southern Ross Sea, adult and subadult Antarctic toothfish (*Dissostichus mawsoni*) [47], we propose that an increase has occurred in a principal prey species of both predators, Antarctic silverfish (*Pleuragramma antarcticum*). Over the southern shelf, Antarctic silverfish are a major prey of both Adélie penguins and Antarctic toothfish (cf. [48], [49]). Consuming silverfish is key to large size and maximum body condition of Ross Island penguin chicks, and thus eventual post-fledging survival (Whitehead et al. unpubl. data). Such a change in survival could explain the population increase, a subject currently being explored (Dugger et al., unpubl. data).

### Trends in the Northern Victoria Land Population: More Physics

It is unfortunate that colony assessment became so sparse for northern Victoria Land after 2000. Before then, trends appear to be approximately similar to the southern colonies. Since then, it is impossible to judge trends, and it appears that the slow movement of the B-15, C-16 and C-19 icebergs along the Victoria Land coast negatively affected the northern colonies, coincidently in most of the years when counts were done. The mega-iceberg C-19 delayed sea-ice breakout offshore of most of the northern Victoria Land colonies as it made its way north in Spring 2002. This would help to explain the low counts that year, with the ice having retarded or discouraged penguin arrival in time for egg laying (and counting). Similarly, B-15J (the largest piece remaining from B-15A, after it broke up further) and C-16 appear to have blocked the exit of sea ice offshore of northern colonies as they made their way north in 2006 (another year of low counts). It would have been interesting to have had colony counts from Possession, Sven Foyn and Cape Adare colonies in 2005, as the largest part of B-15 spent several summer months offshore of those colonies in late 2005 [50] (it passed by the more southerly Victoria Land colonies well before penguins would have been undertaking their spring migration and arrival at colonies and so likely had no effect then). The effect on packing the sea ice against the coast by one small, B-15A, piece off Cape Hallett in 2005 can be seen in fig. 4 in Lyver *et al*. [51], potentially resulting in alteration of the foraging area. We did not have a colony count that year, and the low colony size in 2006, for whatever reason, seems to have been temporary. Indeed, a report based on an aerial survey in 2009 to revise the Cape Hallett Management Plan indicated that breeding pairs were far more numerous than when we did our last survey in 2006 [52]. Using a combination of oblique photographs from an aircraft flying just outside colony boundaries and ground-truthing, the surveyors estimated ∼64,000 breeding pairs, or a 2½-fold difference from the numbers counted in 2006. We cannot explain the difference. However, lessons learned at Ross Island, and especially Cape Royds, suggest that mega-iceberg presence, including lagged effects (Dugger *et al*. unpubl. data), could well apply.

## Conclusion

As a sea-ice obligate, the Adélie penguin generally exists in areas of intermediate sea-ice concentration (∼15% is ideal [53]), where there is adequate ice for resting but not so much that individuals incur additional energetic cost associated with walking great distances to access open water [12], [54]. We saw the effects of too much ice in the trends of both the southern and northern Victoria Land colonies, especially with the mega-iceberg natural experiments. Another experiment is taking place elsewhere in Antarctica, as sea ice disappears and so have Adélie penguins [15], [16], [55], [56]. On the basis of that information, predictions related to changes in Adélie penguin populations, size and distribution, as a function of sea-ice presence and persistence, indicate that 75% of Adélie penguin colonies (70% of the breeding population) north of 70° S will decrease or disappear by 2050 owing to the disappearance of sea ice but colonies may grow or be founded at high latitude where sea ice is loosening [9]. All of the Ross Sea colonies reported in the present study, however, are situated south of 70° S, and may well be the last to benefit from sea-ice presence if current climate trends continue.
